# Supplementary figures and images for: Ciliary muscles contraction leads to axial length extension——The possible initiating factor for myopia
Source: PLoS One. 2024 Apr 16;19(4):e0301844. doi: 10.1371/journal.pone.0301844 (PMC11020782; doi:10.1371/journal.pone.0301844)

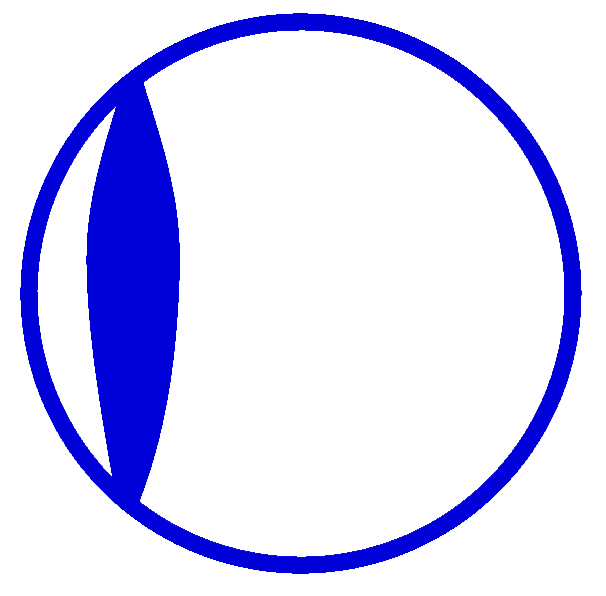

Supplement: S1 Fig — (GIF) [file pone.0301844.s002.gif]
